# Impact of different fracture types in the pyriform buttress area on nasal airway function

**DOI:** 10.1007/s00405-023-08290-5

**Published:** 2023-10-20

**Authors:** Zhongying Wang, Dong Chen

**Affiliations:** 1 Department of Otolaryngology-Head and Neck Surgery, Shanghai Ninth People’s Hospital, Shanghai Jiao Tong University School of Medicine, Shanghai, China; 2 Ear Institute, Shanghai Jiao Tong University School of Medicine, Shanghai, China; 3 Shanghai Key Laboratory of Translational Medicine On Ear and Nose Diseases, Shanghai, China

**Keywords:** Fracture types, Nasal airway function, Nasal obstruction symptom evaluation scale, Pyriform buttress area fracture, Acoustic rhinometry, Rhinomanometry

## Abstract

**Introduction:**

Fractures in the pyriform buttress area adversely affect facial appearance and nasal airway patency. Nasal airway function has received less attention than aesthetic problems in the literature. This retrospective study classified the different fracture types in this area and determined their impact on nasal airway function.

**Mathods:**

Three-dimensional computed tomography images of patients with fractures in the pyriform buttress area were analyzed to identify the exact fracture pattern. The nasal airway functions were evaluated and compared between patients with different fracture patterns using acoustic rhinometry, rhinomanometry, and the nasal obstruction symptom evaluation scale.

**Results:**

Overall, 47 patients, including 16 with type I fractures (high fracture line; group I), 16 with type II fractures (intermediate fracture line; group II), and 15 with type III fractures (low fracture line; group III), were included in the study. The mean minimal cross-sectional area (MCA), total nasal inspiratory resistance (Tri) and total nasal expiratory resistance (Tre) of group I were 0.51 ± 0.06 cm^2^, 1.67 ± 0.11 kPa L^−1^ s^−1^, and 1.66 ± 0.12 kPa L^−1^ s^−1^, respectively; those of group II were 0.48 ± 0.07 cm^2^, 1.89 ± 0.15 kPa L^−1^ s^−1^, and 1.88 ± 0.14 kPa L^−1^ s^−1^, respectively; and those of group III were 0.36 ± 0.04 cm^2^, 1.94 ± 0.21 kPa L^−1^ s^−1^, and 2.01 ± 0.34 kPa L^−1^ s^−1^, respectively. The nasal obstruction symptom evaluation (NOSE) scale scores for groups I, II, and III were 7.188, 9.813, and 13.27, respectively.

**Conclusion:**

Therefore, the severity of the nasal airway obstruction depends on the displacement of the fractured bones in patients with fractures in the pyriform buttress area. The most profound nasal obstruction occurs in patients with the lowest fracture line.

## Introduction

The frontal processes of the maxilla and nasal bone form an essential part of the midface. Over 66% of patients with nasal trauma have fractures in this area [1]. The anatomical position of the frontal process of the maxilla is relatively unique and involves the lateral edge of the pyriform foramen, maxilla, and infraorbital edge. Previous studies have described cases in which the displaced segments do not extend to the fronto-maxillary suture as medial maxillary fractures [2, 3]. When the cephalic border of the affected bony segments is higher, the fracture is classified as a type I naso-orbito-ethmoid fracture [4]. Fractures in this area have severe aesthetic implications, including facial asymmetry and nasal and infraorbital rim deformities. In addition to aesthetic problems, fractured bony fragments in the pyriform buttress cause the medial collapse of the nasal vault, leading to nasal airway obstruction. However, few studies regarding the nasal airway function after such fractures have been reported [5, 6].

The nasal airway resistance can be affected by the structures of the upper lateral cartilage, nasal septum, and inferior turbinate. The positions of the fractured pieces determine the nasal airway function. A high fracture line in the piriform buttress area results in the collapse of the upper-third of the nasal vault. A fracture line through the bone attachment to the middle turbinate leads to the collapse of two-thirds of the lateral wall, as well as the middle turbinate. The lower the fracture line along the pyriform rim, the greater the displacement of the ipsilateral nasal vault. When the fracture line reaches the bottom of the pyriform rim, the nasal airway obstruction may be severe even when the bony displacement is minimal [7].

This retrospective study analyzed the impact of different fracture types of the pyriform buttress area on nasal airway function. The patients’ nasal airway functions were examined both objectively and subjectively.

## Materials and methods

This single-center, retrospective study included 47 patients who experienced pyriform buttress fractures between 2016 and 2022. The history of nasal airway patency, direction of nasal blows, and occurrence of epistaxis were recorded for each patient. Swelling, mucosal tearing, intranasal hematoma, depression, and septal deviation at the injury site were recorded. Three-dimensional computed tomography (CT) images of the patients were analyzed to identify the exact fracture patterns according to the degree of comminution, displacement, and septal fracture. The nasal airway function was evaluated using acoustic rhinometry, rhinomanometry, and the NOSE scale. This study was approved by the Institutional Ethics Committee of our hospital (Institutional Review Board number: SH9H-2023-T160-1) and conducted according to the Declaration of Helsinki.

Patients with nasal obstruction before the injury due to chronic allergic rhinitis or septal deviation were excluded as were those who had previously undergone surgery on the nose or paranasal sinuses. Patients who refused to participate were also excluded.

### Fracture type in the pyriform buttress

**Fig. 1:**
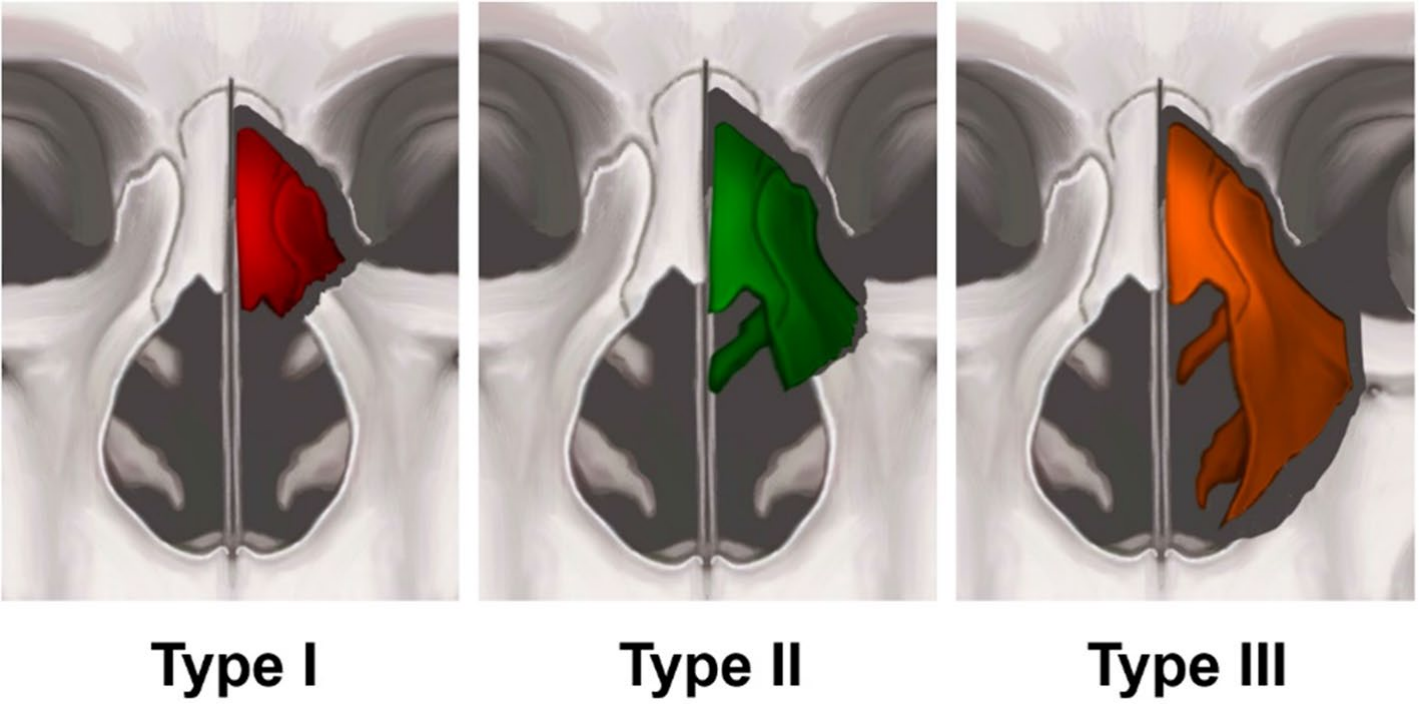
The three fracture types of the pyriform buttress area are shown

The fracture pattern was classified based on the characteristics identified on the CT images (Fig. 1). The patients were divided into groups based on the fracture types, as follows: group I, type I fractures (high fracture line which passes through the upper third of the nasal pyramid, resulting in the collapse of the upper third of the nasal vault); group II, type II fractures (intermediate fracture line, which passes through the bone attachment to the middle turbinate, leading to the collapse of two-thirds of the nasal vault and middle turbinate); and group III, type III fractures (low fracture line, which extends to the bottom of the pyriform rim, resulting in the collapse of the ipsilateral nasal wall, middle turbinate, and inferior turbinate).

### Acoustic rhinometry

An Eccovision Acoustic Rhinometer (HOOD Laboratories, Pembroke, MA, USA) was used to objectively evaluate the internal nasal airway. This anatomical assessment is highly sensitive to subtle changes in the nasal valve area as determined by calculating the MCA. An external nasal adapter was selected to achieve a proper fit for each patient, and a thin layer of ointment was applied to prevent acoustic leakage between the nostril and adapter. The nose adaptor was positioned carefully such that the nasal valve anatomy was not distorted. Before the assessment, the nose was decongested using a topical decongestant to eliminate physiological variations.

### Rhinomanometry

Rhinomanometry is a functional test of nasal aerodynamics that measures nasal airflow and the pressure gradient between the nasopharynx and nostril, allowing for the determination of nasal airway resistance. While the airflow through one side of the nose was measured, the contralateral cavity was sealed and monitored using a small tube to measure nasopharyngeal pressure. Therefore, the pressure curves and nasal resistance were determined separately for each nasal passage, and the total was then calculated. Before the assessment, a topical decongestant is used to eliminate physiological variations of the nasal mucosa.

### NOSE scale

The NOSE scale is a brief questionnaire that assesses the extent of the patient-perceived nasal obstruction before and after medical or surgical treatment that has been validated in previous studies [8–10]. The scale ranges from 0 to 20. All patients in this study responded to the NOSE scale questionnaire.

### Statistical analyses

Data are presented as the mean and standard deviation. The data were compared between the groups using the unpaired Student’s *t* test. The level of significance was set at *p* < 0.05.

## Results

A total of 47 patients (mean age 32.5 years; range 21–65 years), including 31 (66%) men and 16 (34%) women, completed this study. The average period between the facial injury and nasal airway test was 13 (range 12–17) days. Groups I and II each included 16 patients, while group III included 15.

**Fig. 2:**
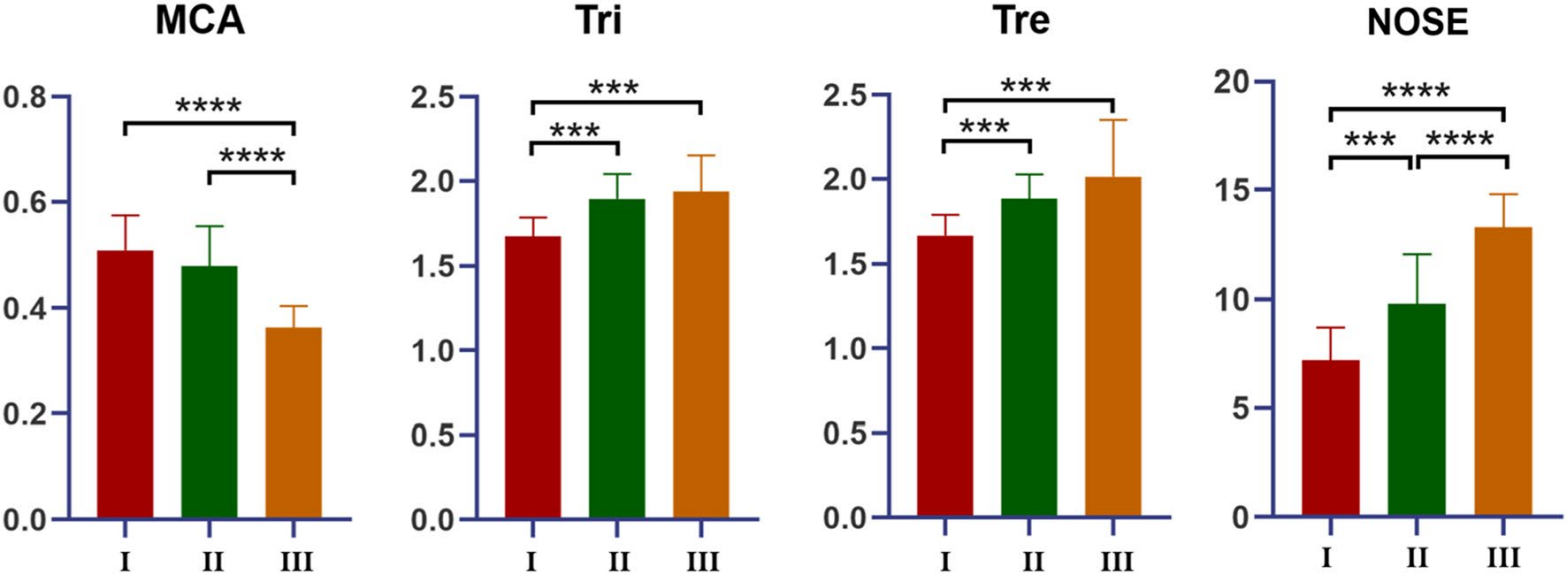
AR results and NOSE scale scores. The MCA of group III was the lowest among three groups (*p* < 0.0001). The MCA of group I was higher than that of group II (*p* = 0.233). The Tri and Tre of group I were the lowest among three groups (*p* < 0.001). The Tri and Tre of group III were lower than that of group II (*p* = 0.507, *p* = 0.166). The NOSE score was significantly lower in group I (7.118) than in groups II (9.813) and III (13.27) (*p* < 0.001). The score of group III was the highest among them (*p* < 0.001)

The MCA of group III (0.36 ± 0.04 cm^2^) was significantly lower than that of groups I (0.51 ± 0.06 cm^2^) and II (0.48 ± 0.07 cm^2^) (*p* < 0.0001). The MCA was not significantly different between groups I and II (*p* = 0.233). The Tri was significantly lower in group I (1.67 ± 0.11 kPa L^−1^ s^−1^) than in groups II (1.89 ± 0.15 kPa L^−1^ s^−1^) and III (1.94 ± 0.21 kPa L^−1^ s^−1^) (*p* < 0.001), although it was not significantly different between groups II and III (*p* = 0.507). The Tre was significantly lower in group I (1.66 ± 0.12 kPa L^−1^ s^−1^) than in groups II (1.88 ± 0.14 kPa L^−1^ s^−1^) and III (2.01 ± 0.34 kPa L^−1^ s^−1^) (*p* < 0.001), although it was not significantly different between groups II and III (*p* = 0.166) (Fig. 2 and Table 1).

**Table 1:** Acoustic rhinometry and rhinomanometry results

Group	*n*	MCA (cm^2^)	Tri (kPa L^–1^ s^–1^)	Tre (kPa L^–1^ s^–1^)
I	16	0.51 ± 0.06	1.67 ± 0.11	1.66 ± 0.12
II	16	0.48 ± 0.07	1.89 ± 0.15	1.88 ± 0.14
III	15	0.36 ± 0.04	1.94 ± 0.21	2.01 ± 0.34

*MCA* minimal cross-sectional area, *Tri* total nasal inspiratory resistance, *Tre* total nasal expiratory resistance

The NOSE score was significantly lower in group I (7.118) than in groups II (9.813) and III (13.27) (*p* < 0.001) (Fig. 2). Difference between group II and III was also significant (*p* < 0.001).

Here we introduce 4 typical cases with pyriform buttress area fractures. 3D-CT images of them were analyzed to identify the exact fracture pattern which affects nasal airway differently.

### Case 1

**Fig. 3:**
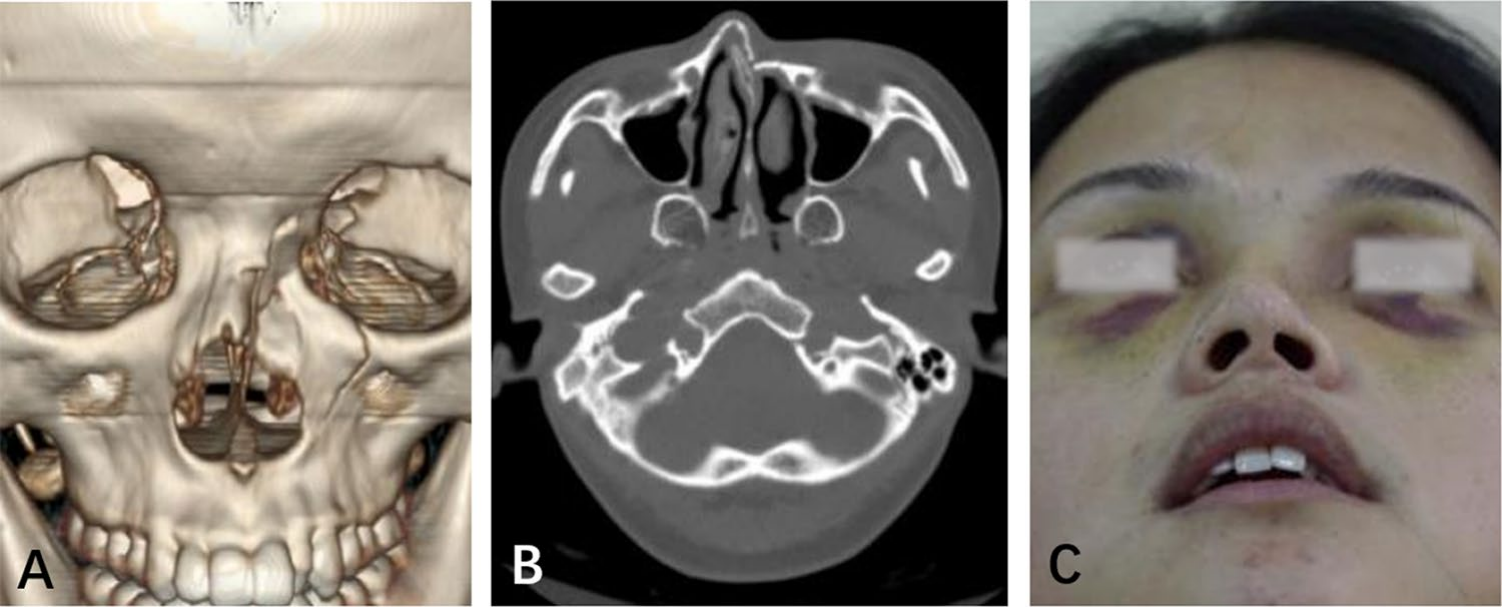
Type I fracture in the left pyriform buttress area. Three-dimensional and horizontal computed tomography images reveal the collapse of the left upper third of the nasal vault (**A**, **B**). **A** slightly asymmetry of the nostrils was observed from the head up view (**C**)

A 21-year-old woman was hit on the left side of her face. She presented with bruising around the eyes, nasal distortion, and epistaxis. CT images demonstrated that her left pyriform buttress had a type I fracture, causing the collapse of the upper third of the nasal vault in the left nasal cavity (Fig. 3A, B). A slight asymmetry of her nostrils was observed from the head up view (Fig. 3C).

### Case 2

**Fig. 4:**
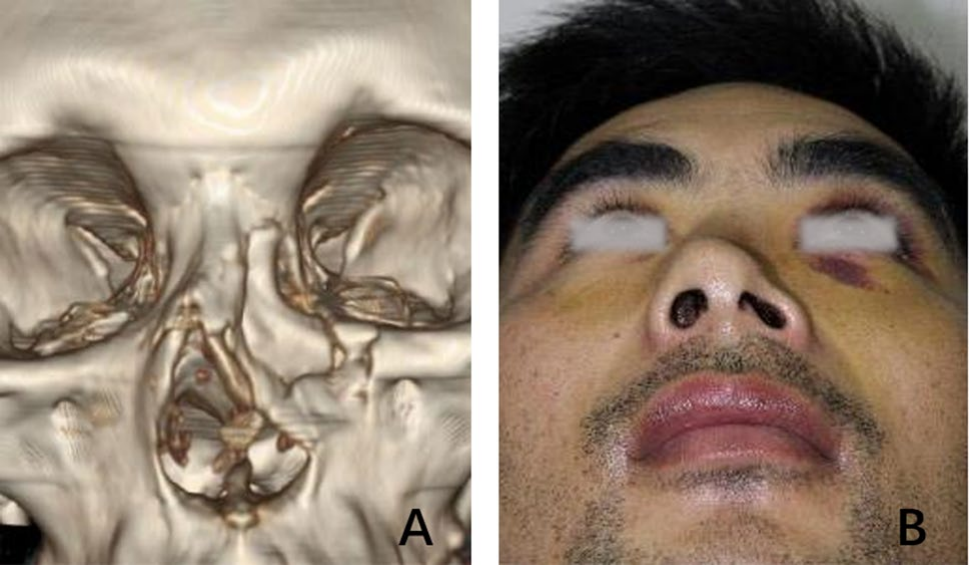
Comminuted type II fracture. Three-dimensional and horizontal computed tomography images reveal the collapse of the left two-thirds of the nasal vault and middle turbinate (**A**). An obvious asymmetry of the nostrils was noticed (**B**)

A 34-year-old man experienced facial injury in a car accident. He had bruising around the eyes, facial and nasal deformities, and nasal obstruction. CT images demonstrated that his left pyriform buttress had comminuted type II fractures, causing the collapse of over two-thirds of the nasal wall (Fig. 4A). An obvious asymmetry of his nostrils was noted on the head up view (Fig. 4B).

### Case 3

**Fig. 5:**
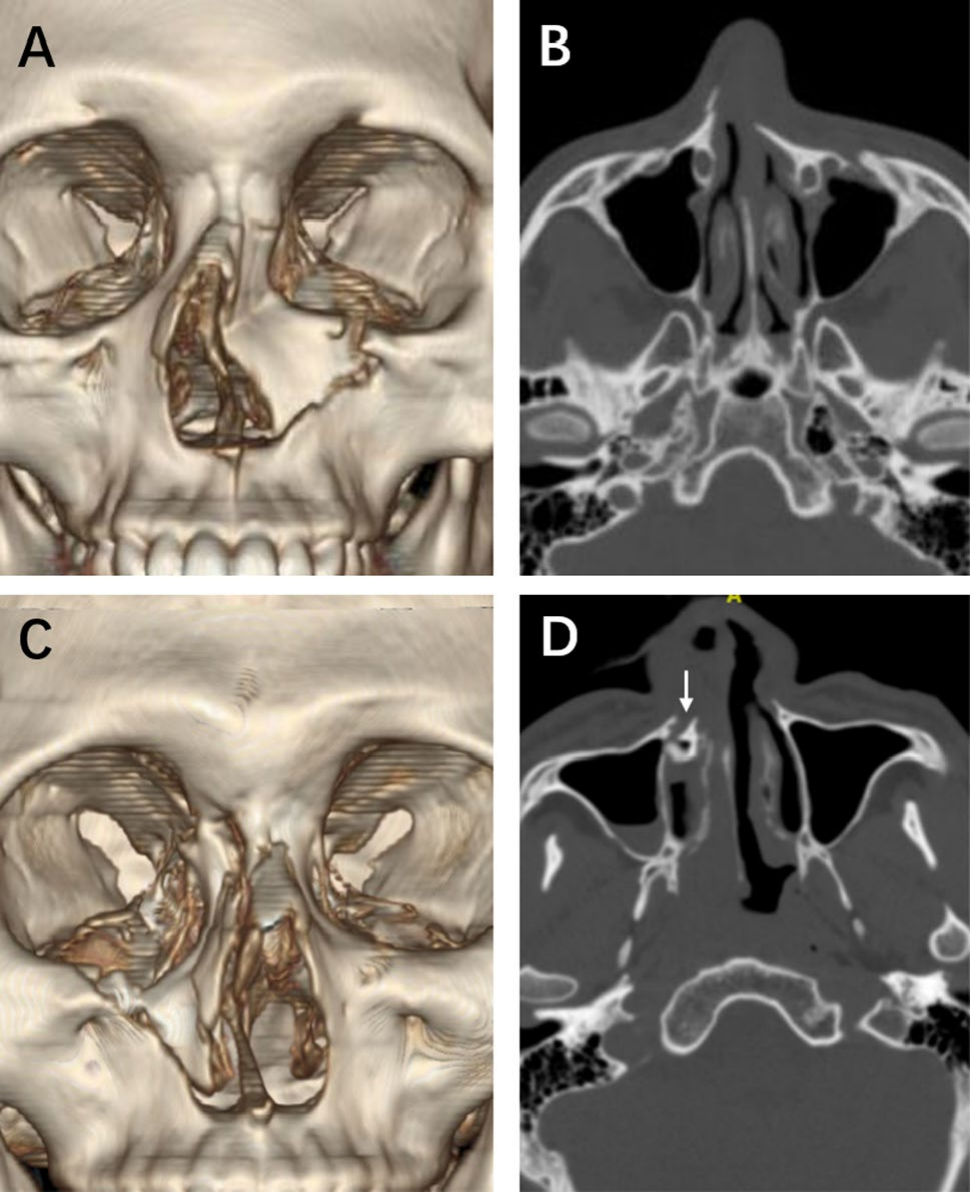
Type III fractures. CT images demonstrated a 17-year-old boy’s left pyriform buttress had a type III fracture, which caused the collapse of the ipsilateral nasal wall (**A**). The left nasal airway was completely blocked by the fractured bone fragment (**B**). Another young boy had comminuted type III fractures, with collapsing of the ipsilateral nasal wall and inferior turbinate (**C**, **D**). The right bony nasolacrimal canal was also fractured (**D**, white arrow)

A 17-year-old boy experienced a facial injury following a car accident. He had facial and nasal deformities and severe nasal obstruction. CT images demonstrated that his left pyriform buttress had a type III fracture, which caused the collapse of the ipsilateral nasal wall (Fig. 5A). The left nasal airway was completely blocked by the fractured piece (Fig. 5B).

### Case 4

A 15-year-old boy experienced a facial injury in a motorcycle accident. The patient had facial and nasal deformities, severe nasal obstruction, and epiphora. CT images demonstrated that his right pyriform buttress had comminuted type III fractures, causing the collapse of the ipsilateral nasal wall and inferior turbinate (Fig. 5C, D). The right bony nasolacrimal canal was fractured (Fig. 5D, white arrow).

## Discussion

The frontal process of the maxilla is an extension of the maxilla and is part of the nasal pyramid structure. Improper treatment of fractures in this area affects the aesthetic shape and airway function. Although intuitively simple and convenient, nasal airway assessment is often ignored, leading to dissatisfactory results. Preexisting respiratory functional disorders are a major factor in secondary rhinoplasty [11, 12]. Some revision surgeries may be avoided with proper evaluation of the nasal function conducted at the time of the fracture. Familiarity with the nasal anatomy, physiology, and pathology allows surgeons to manage these fractures appropriately.

The nasal cavity is the first part of the respiratory tract and provides the majority of the resistance within the respiratory tract. During nasal breathing, over 50% of the respiratory resistance is caused by the nose [13]. Previous studies have suggested that the nasal valve plays a more important role in nasal airway function than the nasal septum [14–16]. The nasal valve area is composed of the inferior turbinate head, septum, upper lateral cartilage, and surrounding soft tissue. Minor changes in this area may significantly affect nasal breathing. Fractures in the pyriform buttress area involving displaced bony segments cause nasal vault collapse, leading to anatomical variations in the nasal valve area.

The patients in this study presented immediately after their injury. They were determined to have persistent nasal obstruction after 10 days during which the swelling of the mucosa was permitted to subside. The NOSE scores were significantly different between the groups in this study. However, quantification of the subjective sensation of nasal obstruction is difficult as patient perceptions vary. Therefore, objective nasal assessments were also conducted.

AR has been used to measure the airway resistance and to assess nasal obstruction. A topical decongestant was used before the assessment to help eliminate the effects of mucosa factors. The MCA, MCA distance, and volume were measured, although only the MCA was evaluated in this study as it is the most sensitive to changes in the nasal structure. In this study, the MCA was the smallest in patients with the lowest fracture lines. Lower fracture lines were associated with collapse of the upper lateral cartilage and medialization of the inferior turbinate. Excluding the effect of the septum, the two components of the valve area changed, resulting in a decrease in the MCA. The MCA was not significantly different between groups I and II in this study, which may be due to the fact that the fracture lines were higher on the nasal pyramid, leading to a change in only one component of the nasal valve structure.

In addition, the Tri and Tre were the lowest among patients in group I, which is consistent with the AR results. These results suggest that patients with high fracture lines have less disruption in the valve area. The Tri and Tre were not significantly different between groups II and III, which may be due to the small sample size of this study or measurements that were not sensitive enough to differentiate between the groups.

This study presents four individual cases of unilateral fractures around the frontal process of the maxilla. The CT images show that the isolated fragments were displaced medially and downward, causing medialization of the lateral nasal wall and nasal obstruction. The patients in cases 1–4 had typical fracture styles, indicating high-, intermediate-, and low-fracture lines. Patients 2 and 4 had comminuted fractures. The nasal airway function appears to be related to the position of the fracture rather than the severity of the fracture.

We propose that the fractures in the pyriform buttress area are very unique which affects the facial appearance and nasal airway function simultaneously. While handling type II and type III fractures, a combination of gingivobuccal and subciliary incisions will make a good view and exposure to the fractured area. Type I fracture with obvious bony displacement could be repaired through transnasal incision. Type I fracture with very slight bony displacement and no aesthetic and functional impairment has no need for surgery.

This study was limited by its small sample size of 47 patients. In addition, this was a retrospective study and all assessments were limited to a single teaching hospital, although our patients were from different provinces across the country. Furthermore, the patients’ septum was not analyzed in this study as the included patients had no nasal obstruction before their injuries.

In summary, different fracture patterns of the pyriform buttress area result in nasal obstruction, and the severity of obstruction depends on the displacement of the fractured pieces. The most profound nasal obstruction occurs in patients with the lowest fracture lines.
